# A Study on the Sound Absorption Properties of Mycelium-Based Composites Cultivated on Waste Paper-Based Substrates

**DOI:** 10.3390/biomimetics7030100

**Published:** 2022-07-22

**Authors:** Natalie Walter, Benay Gürsoy

**Affiliations:** Department of Architecture, Penn State University, University Park, PA 16802, USA; bug61@psu.edu

**Keywords:** mycelium, acoustic materials, bio-fabrication, sound absorption

## Abstract

Mycelium-based composites have the potential to replace petrochemical-based materials within architectural systems and can propose biodegradable alternatives to synthetic sound absorbing materials. Sound absorbing materials help improve acoustic comfort, which in turn benefit our health and productivity. Mycelium-based composites are novel materials that result when mycelium, the vegetative root of fungi, is grown on agricultural plant-based residues. This research presents a material study that explores how substrate variants and fabrication methods affect the sound absorption properties of mycelium-based composites grown on paper-based waste substrate materials. Samples were grown using *Pleurotus ostreatus* fungi species on waste cardboard, paper, and newsprint substrates of varying processing techniques. Measurements of the normal-incidence sound absorption coefficient were presented and analyzed. This paper outlines two consecutive acoustic tests: the first round of experimentation gathered broad comparative data, useful for selecting materials for sound absorption purposes. The second acoustic test built on the results of the first, collecting more specific performance data and assessing material variability. The results of this study display that cardboard-based mycelium materials perform well acoustically and structurally and could successfully be used in acoustic panels.

## 1. Introduction

Increasing urban populations, scarce resources, and climate change will force a paradigm shift in our material use and approaches to construction. Our current framework of construction is unsustainable; we rely on fleeting systems of resource extraction, waste management, and energy consumption. By relying on man-made polymers and petroleum-based components in our built environment, our building materials either cannot naturally decay or take centuries in a landfill to degrade. Biodegradable materials and biologically derived materials present an alternative to this traditional construction framework. Mycelium-based composites, a bio-material derived from fungi, have the potential to successfully replace plastic-based materials in our building systems without the extraction of non-renewable resources. Instead, mycelium, the vegetative root of fungi, is grown on agricultural plant-based residues, resulting in a new compound material. This research aims to further understand the characteristics of the material and the potential for implementation as acoustic architectural components. Specifically, this research began with systematic material tests, assessing the acoustic properties of mycelium-based components grown on local and accessible paper-based waste products. These material tests then inform the development of mycelium-based sound absorption panels. Using *Pleurotus ostreatus* fungi species, commonly known as the oyster mushroom, this research tested how substrate variants and fabrication methods affect acoustic absorption.

### 1.1. Noise Control through Sound Absorption

Exposure to prolonged environmental noise is associated with several negative effects that can be mitigated with proper sound treatments. Chepesiuk addresses the health problems associated with hazardous noise, including tinnitus, elevated blood pressure, cardiovascular constriction, and hearing loss [1]. These effects, in turn, lead to social handicaps, reduced workplace productivity, and decreased student–teacher communication. The Centers for Disease Control and Prevention (CDC) even declares that “occupational hearing loss is one of the most common work-related illnesses in the United States” [2]. Addressing this problem requires the implementation of noise control treatments in architectural systems to reduce the negative effects of noise.

Noise control and architectural acoustics are a growing sector of the design field, given the importance of maintaining acoustic comfort. Aletta and Kang argue that while noise can be hazardous, the pursuit of “silence” from a health standpoint is not what defines a successful acoustic environment [3]. They point out challenges in architectural acoustics but suggest that we move away from total noise control and instead embrace a certain threshold of environmental sound. Therefore, thoughtful consideration must be made to regulate acoustic quality rather than just reducing all sound.

Regulating interior acoustic quality is performed through environmental assessment and sound treatment, pending the spatial and programmatic requirements. All building materials either reflect, transmit, or absorb incident sound, and thus to manage acoustic comfort, materiality must be designed with acoustic intent [4].

#### 1.1.1. Sound Absorption

Sound absorption is one method of acoustic treatment in which the energy of a sound wave is converted into low-grade heat, reducing the strength of reflected sound [4]. This reduces the amount of sound perceived as well as the effects of acoustic discomfort. Sound absorptive materials have many different applications within architectural, studio, automotive, and industrial acoustics. They can be used as interior lining in vehicles, aircraft, ducts, industrial equipment, and buildings/interiors. These materials are notably used within performance spaces to control unwanted echo, work environments to quiet the reverberant field, and restaurants to improve users’ communication [5]. A measurement of a material’s sound absorption is called the sound absorption coefficient, which is the ratio of energy absorbed to the incident energy. The higher the sound absorption coefficient, the more absorptive the material [4].

There is a need to develop sustainable alternatives for conventional synthetic sound-absorbing materials (i.e., glass wool, stone wool, and polystyrene). Both Arenas and Sakagami [6] and Desarnaulds et al. [7] address the environmental impacts of conventional sound-absorbing materials. Arenas and Sakagami mentioned that sound absorbing materials began with asbestos-based materials but were replaced with mineral-based fibrous materials once asbestos was linked to human health hazards. These fibers are most commonly made from glass and rock wool fibers, but their use is associated with negative environmental effects. The researchers suggest the use of sustainable alternatives, such as “eco-materials elaborated from residues” [6]. Desarnaulds et al. added to this by assessing the environmental performance of sustainable acoustic materials [7]. In this article, they specified that glass and rock wools are unsustainable because they are disposed of in a non-inert waste landfill. They also release airborne fibers that are harmful to contractors, laborers, and future occupants.

#### 1.1.2. Factors Influencing Sound Absorption

Sound absorptive materials are generally fibrous or porous in nature. Their absorption behavior is dependent on physical material characteristics, such as the following: Fiber size, porosity, material thickness, and material density [5].

*Fiber Size:* Fiber diameter affects sound absorption because of the fiber’s movement when sound waves travel through the material. Fibers act as frictional elements, which convert sound energy into heat as they move. Thinner fibers have a higher sound absorption coefficient for two reasons. First, thin fibers move more easily than thicker fibers. Second, more fibers are needed to reach the same volume density as a material with thicker fibers, which creates more tortuous paths for sound waves, thus increasing airflow resistance [8]. Thus, having many thin fibers in a material rather than a few thick fibers creates greater frictional resistance.

*Porosity:* Porosity deals with the number, size, and type of pores/voids existing in a material through which sound waves travel through and become dampened. When sound waves enter pores, the air molecules within the channels vibrate, converting part of the sound energy into heat [9]. Continuous channels are more successful at absorbing sound than shorter, closed pores.

*Material’s Thickness:* The thickness of a sound-absorbing material has a direct relationship with low-frequency sounds (100–2000 Hz), while it has no effect on high-frequency sounds. As the material becomes thicker, the sound absorption increases. Studies show that effective sound absorption for low-frequency sounds is achieved when the thickness is approximately one-tenth of the wavelength of the incident sound [5].

*Material’s Density:* The sound absorption coefficient increases for middle and high-frequency sounds as the density of the material increases. Less dense materials absorb low frequencies (500 Hz), while denser structures absorb higher frequencies (2000 Hz) [5].

The relationship between material characteristics and acoustic performance is also relevant with regard to musical instruments. Wegst [10] addressed why the physiological properties of bamboo and wood make them ideal materials for instrument manufacturing. An important point made is that the loss coefficient (acoustic energy dissipated due to friction) is dependent on the temperature and moisture content within a sample.

Understanding the physical material characteristics that determine acoustic performance is relevant to this research because the growth factors of mycelium-based composites can be curated to achieve optimal acoustic performance. Since mycelium-based composites characteristics are highly variable, understanding what outcome is preferred enables narrowing down the growth parameters.

#### 1.1.3. Testing Sound Absorption

Testing sound absorption can be performed using different methods depending on the desired result. In order to test the sound absorption of a specific material, an impedance tube is often used. The two-microphone transfer-function method is a common method when using an impedance tube. This is when a sound source sends broadband sound waves at a sample, which reflect off the sample. The sound waves generate a pattern of forward and backward traveling waves inside the tube. Digital frequency analyzers then measure the sound pressure at specific locations to determine the sound absorption and acoustic impedance of the material.

### 1.2. Mycelium-Based Composites as Biodegradable Alternatives for Sound Absorption

The construction industry generates a significant amount of waste with undeniable negative environmental impacts. The use of biodegradable materials as building components can reduce the amount of building waste generated and the ensuing environmental consequences. Transporting waste is associated with resource consumption and pollution emissions, landfills are associated with land use and ground contamination, and waste incineration produces contaminated ash, air pollution, and greenhouse gas emissions [11]. According to the Environmental Protection Agency (EPA), approximately 600 million tons of construction and demolition debris were generated in 2018, which amounts to more than twice the amount of municipal solid waste generated in the same year [12]. There is a clear need to reduce the amount of waste generated from building construction and demolition, and biodegradable materials offer a low waste alternative.

#### 1.2.1. Cultivating Mycelium-Based Composites

**Figure 1:**
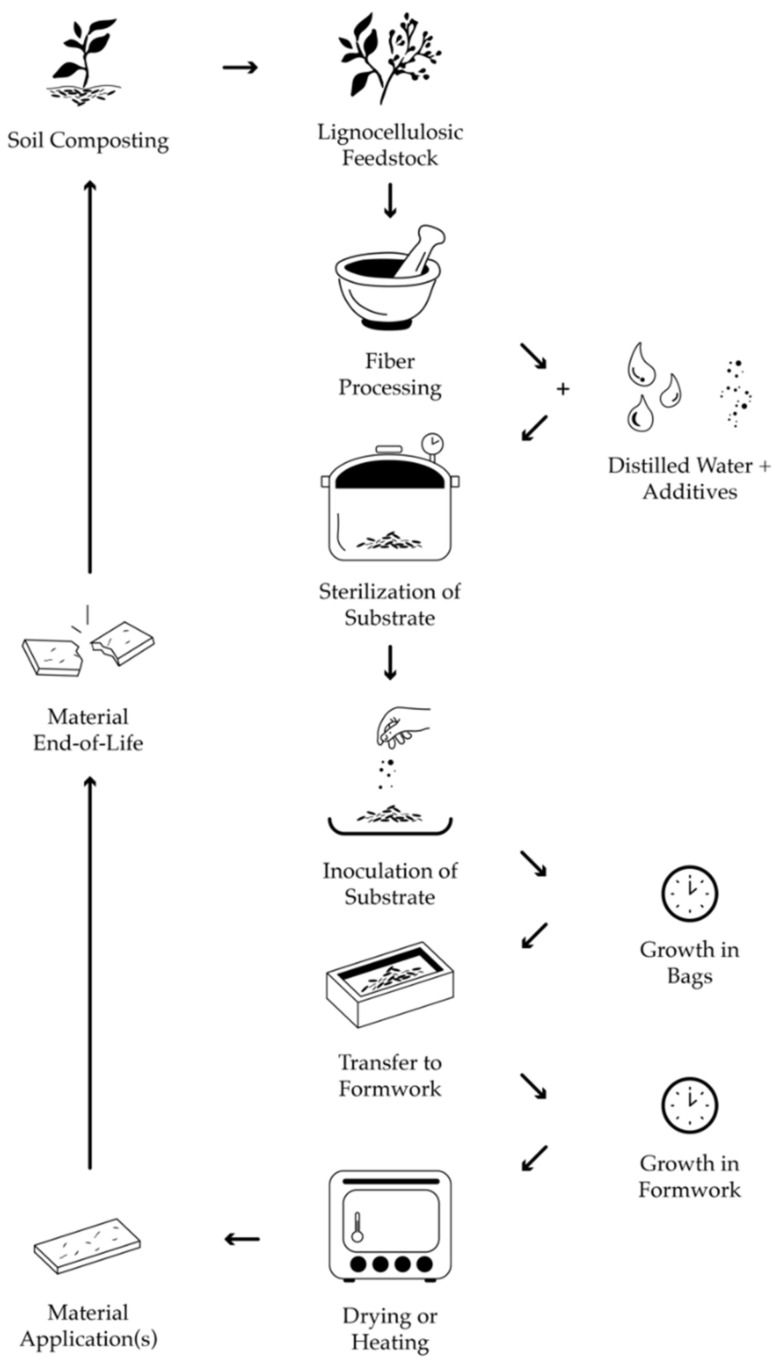
Typical Stages of Mycelium-Based Composite Cultivation.

Mycelium-based composites result when fungal growth is stopped during colonization of the substrate, and a resulting compound material is created [13]. Mycelium grows in search of food and spreads through the substrate in a network colony. During this growth, mycelium produces enzymes that convert the substrates’ biomass into nutrients while simultaneously binding the substrate. The organic matter decomposes over time as the plant polymers are replaced with fungal biomass. Fabrication of mycelium-based composites involves the growth of mycelium on organic substrates. The composites’ properties and performance are highly variable; factors include fungal species, substrate type, environmental conditions during growth (temperature, humidity), and forming/processing techniques [14]. The resulting materials differ immensely in their density, tensile and compressive strength, morphology, and insulative/acoustic performances [15]. Figure 1 illustrates the typical stages of mycelium-based composite cultivation.

There is a growing field of knowledge on mycelium-based composites as more researchers are testing the characteristics of different growth methodologies. It is important to note that because the material constitution and mechanical properties of mycelium-based composites vary immensely, it is difficult to establish set protocols for growth and fabrication methods. The compressive strength of one composite material, for example, may be drastically different than another composite because of the different growth protocols. That said, below are some prominent experiments that assess the physical, chemical, and mechanical properties of mycelium-based composites.

Appels et al. [16] experimented with the growth and fabrication techniques of mycelium-based composites by growing *Trametes multicolor* and *Pleurotus ostreatus* on beech sawdust and rapeseed straw. Appels et al. found that the different fungal strains and substrate compositions cause differing mechanical and physical characteristics of the resulting composite. One finding, for example, is that *Trametes multicolor* grown on rapeseed straw resulted in flexible and soft skin, while *Pleurotus ostreatus* also grown on rapeseed straw resulted in firm and rough skin.

Elsacker et al. [15] grew *Trametes versicolor* on five different fiber types (hemp, flax, flax waste, softwood, and straw). They also varied the fiber processing techniques into four categories: loose, chopped, dust, pre-compressed, and tow. The resulting materials were then tested for dry density, Young’s modulus, compressive stiffness, stress–strain curves, thermal conductivity, and water absorption rate. One finding that Elsacker discovered was that the mechanical properties of the composites are dependent on fiber types. The fiber condition (loose vs. chopped) had a large impact on the compressive stiffness, and the samples grown were dense.

#### 1.2.2. Mycelium-Based Composites as Sound Absorbers

There is limited research and literature existing on the acoustic performance of mycelium-based composites. Moreover, since the resulting material characteristics are variable, the results of one study may not correlate with another. It is difficult to conclude that all mycelium-based composites are successful acoustic absorbers based on the few studies that exist.

Mogu [17] is a company selling mycelium-based interior acoustic wall panels. The company, however, does not disclose its growth methodologies. One prominent study that reported on the experiments on the acoustic properties of mycelium-based composites is [18]. This study tested how substrate variants affect sound absorption. Their substrates were rice straw, hemp pith, kenaf fiber, switch grass, sorghum fiber, cotton bur fiber, and flax shive, and they assessed sound pressure levels. The results found that mycelium-based composites are successful absorbers, but the acoustic performance varies between samples depending on the substrate material. It was also noted that even the low performer, the 100% cotton bur fiber, still yielded higher than 70% acoustic absorption at 1000 Hz. In a subsequent study that built upon this research, the team, instead of testing rigid composites, tested the acoustic properties of mycelium foam [19]. They used *Ganoderma* as the fungal species and a combination of ground corn stover, grain spawn, maltodextrin, and other nutrients as the substrate. They also used a specifically designed growth chamber to grow the foam. These two studies were the main experiments published regarding the acoustic properties of mycelium-based composites, and to gather a further understanding of the acoustic potentials of the mycelium-based composites, more experiments are needed.

Another approach to using mycelium as an acoustic material was seen in the development of the biotech violin [20]. Schwarze and Morris developed a mycelium-based material, coined mycowood, using *Physisporinus vitreus* and *Schizophyllum commune* fungi. This material was developed and manufactured into violins that match the tone of a Stradivarius, an extremely high-quality violin.

Additionally, while not fungal-based, there is a growing field of research regarding alternative natural acoustic materials. Putra et al. [21] analyzed the utilization of natural waste fibers from paddy as an acoustic material. Similarly, Rachman et al. [22] assessed the acoustical performance of a particleboard made of coconut fiber and citric acid solution.

## 2. Materials and Methods

The following experiment consisted of three stages: (1) the cultivation of mycelium-based composites, (2) the assessment of the cultivated samples’ acoustic performance, and (3) the cultivation of mycelium-based acoustic panel prototypes.

Material cultivation began with substrate selection and preparation. The prepared substrates were then sterilized in an autoclave chamber to mitigate contamination. Once sterilized, the materials were inoculated with *Pleurotus ostreatus* spawn. These samples were left to grow in a controlled growth environment, first in autoclavable bags for 12 days and then in sterile formworks for 16 more days. Once grown, the samples were dried and heated in an oven to kill the mycelium and stop the cultivation process. The samples were then shaped to fit into an impedance tube to test for sound absorption. Table 1 shows the samples that were tested in the impedance tube.

The cultivated samples were tested in an impedance tube, following standard ASTM E1050-12, to compare sound absorption in the 500 Hz to 6.4 kHz frequency range.

### 2.1. Cultivation of Mycelium-Based Composites

The following methodology for the growth of these mycelium-based samples was conducted following an initial growth experiment. In the initial experiment, failure to consider material shrinkage resulted in the inability to test for acoustic absorption. The mycelium mixtures were grown in Petri dishes that were the exact size necessary to test for sound absorption. Once dried, they shrunk and warped considerably and would not permit accurate results. The following experiment was executed with shrinkage in mind.

#### 2.1.1. Lignocellulosic Substrate Materials

The selected substrate materials are paper-based waste products, specifically sorted office paper, cardboard, and newsprint. These paper-based materials are all lignocellulosic materials, meaning they provide the lignin and cellulose for fungi to feed. The office paper and cardboard were obtained from recycling bins in the Stuckeman School of Architecture at Penn State University, University Park Campus. The newsprint was similarly obtained from recycling bins across campus and local recycling centers. All materials were sorted to ascertain unsoiled samples.

In order to maintain the cyclical nature of biodegradable materials, the importance of waste and recycled materials was stressed in this study. Thus, strictly local paper-based waste products were used for substrate materials/feedstock. According to the EPA, paper-based materials are largely recycled, yet still, 4.2 million tons of paper were combusted in 2019, making up 12.2 percent of all combusted municipal solid waste (MSW) that year. Additionally, 17.2 million tons of paper-based MSW landed in landfills, making up 11.8 percent of MSW landfilled in 2018 (Environmental Protection Agency, n.d.). This study addressed the accessibility of paper-based waste products and the need to reduce the amount combusted/landfilled.

#### 2.1.2. Substrate Preparation

**Figure 2:**
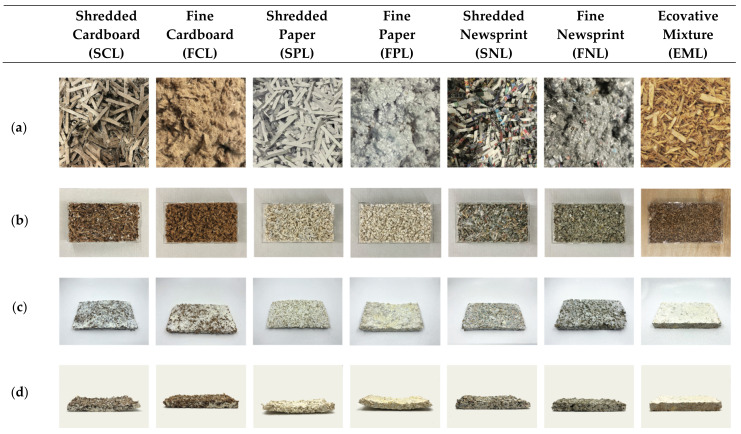
Growth Process of Low-Frequency Samples and Resulting Materials: (**a**) Prepared Substrates (80 mm × 80 mm square); (**b**) Mycelium Mixtures in Formworks (250 mm × 125 mm × 38 mm); (**c**) Composite Materials After Drying (250 mm × 125 mm × 38 mm); (**d**) Composite Materials After Drying—Side View.

Six substrate mixtures were prepared using: (a) shredded cardboard (SCL and SCH), (b) fine cardboard (FCL and FCH), (c) shredded paper (SPL and SPH), (d) fine paper (FPL and FPH), (e) shredded newsprint (SNL and SNH), and (f) fine newsprint (FNL and FNH) seen in Figure 2a. For all samples, the materials (cardboard, newsprint, paper) were first shredded using an office shredder [23]. The three materials were then split in half to make 6 separate sample mixtures, and half of each was ground to make a fine cottony material. All 6 mixtures were supplemented with 10% (*w*/*w*) wheat bran and mixed thoroughly. Wheat bran was used as a supplementary substance to induce mycelial growth and increase cultivation speed by adding nitrogen to the substrate mixtures. The prepared substrates were then adjusted to 65% moisture content by adding water. Each prepared substrate mixture contained 100 g of dry weight material, 185 g of water, and 18 g of wheat bran.

To compare against commercially available mycelium-based composite materials, Ecovative Design’s Grow-It-Yourself Mushroom^®^ Material was also cultivated (EML and EMH) (see Section 2.1.8). The substrate material of these samples was hemp hurd, as seen in Figure 2a.

#### 2.1.3. Sterilization

The 6 substrate mixtures were placed in polypropylene autoclavable bags [24], 200 mm × 125 mm × 480 mm, and stored overnight in a cold room. The bags were then autoclaved for 45 min at 121 °C. This sterilization process assured the substrate was not contaminated with other organisms, making the material unlikely to grow mold. The bags were then cooled down in a clean, room-temperature room overnight.

#### 2.1.4. Inoculation

Each substrate mixture was inoculated with *Pleurotus ostreatus* spawn. The mycelium spawn is purchased from Lambert Spawn [25] (*Strain 123 Pleurotus ostreatus*) in a pre-spawn bag. These prepared bags were made of supplemented cotton seed hulls and straw. A total of 10% of the dry weight of the substrate was added to spawn. The spawn was added directly into the autoclavable bags and thoroughly mixed and compressed. The mycelium was left to grow in the bags for 12 days. The bags are kept in an environmentally controlled growth room, with 99% relative humidity and a temperature of 24 ± 1 degree Celsius.

#### 2.1.5. Cultivation in Formworks

After 12 days of growth in bags, the cultivated mycelium mixtures were transferred to rectangular acrylic formworks, as seen in Figure 2b. Before transferring the cultivated mycelium, the formworks were sterilized with ethanol solution (70%). The transfer from bags to formworks was cautiously performed in a sterile environment. The formworks are then covered with plastic wrap and left to grow for an additional 16 days in the same environmentally controlled growth room.

#### 2.1.6. Heating and Drying

After 16 days, the samples were taken out of the formworks and left to dry with a fan. After two days, the samples were placed in an oven at 90 °C for 24 h, resulting in the rectangular composite materials shown in Figure 2c,d. Drying the samples caused the material to lose 2/3 of its water content and fully kill the mycelium.

#### 2.1.7. Sample Shaping

**Figure 3:**
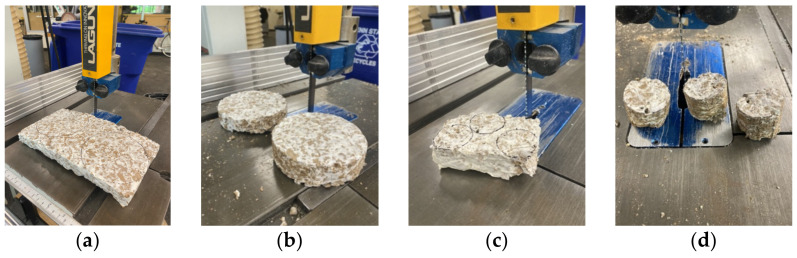
Sample Shaping of Fine Cardboard Material: (**a**) Large Formwork Dried Fine Cardboard Sample; (**b**) FCL Samples (100 mm diameter, 38 mm thickness); (**c**) Small Formwork Dried Fine Cardboard Sample; (**d**) FCH Samples (29 mm diameter, 38 mm thickness).

In order to ascertain whether the samples would fit into the impedance tube to test the sound absorption, the rectangular samples (thickness 38 mm) had to be shaped into 100 mm and 29 mm circles. Therefore, the materials were cut on a band saw, seen in Figure 3, and sanded using a belt sander.

#### 2.1.8. Commercial Mycelium Comparison

In order to compare against commercially available mycelium-based composite mixtures, Ecovative Design’s Grow-It-Yourself Mushroom^®^ Material [26] was grown in the same two formworks and cut to the same sample circles. Ecovative is one of the pioneers in utilizing mycelium-based composites in industrial applications. This start-up began by producing packaging and insulation materials as an alternative to polystyrene-based (Styrofoam) materials and has developed into a large biotechnology company, making myco-leather, mycelium meat alternatives, and beauty industry alternatives [27]. The company sells Grow-It-Yourself bags with their own mycelium mixture. Samples grown using their mixture were also tested in this study (EML and EMH).

### 2.2. Testing and Assessing Sound Absorption of Mycelium-Based Composites

The following experiment outlined two sets of acoustic tests. The first round of tests was useful in selecting appropriate sound-absorbing materials for acoustic panels. The second set of tests builds on the results of the first by testing the best performing materials again using a larger sample size.

#### 2.2.1. Preliminary Testing for Sound Absorption

**Figure 4:**
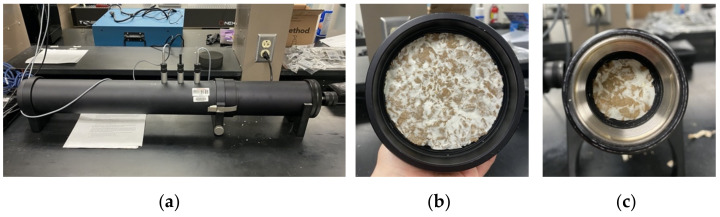
Impedance tube Testing: (**a**) Impedance Tube; (**b**) FCL Sample in the impedance tube; (**c**) FCH sample in the impedance tube.

As a preliminary study, first, two replicates for each of the samples (material thickness: 38 mm) are tested three times using an impedance tube, specifically the two-microphone transfer-function method, illustrated in Figure 4, following the standard ASTM E1050-12. Brüel and Kjær’s Impedance Tube Kit (50 Hz–6.4 kHz) Type 4206 was used in this experiment. Type 4206 consists of:

1. 100 mm diameter tube (large tube)
  a. Frequency range: 50 Hz to 1.6 kHz;
  b. Material sample size requirements: 100 mm diameter, 200 mm max sample length.
2. 29 mm diameter tube (small tube)
  a. Frequency range: 505 Hz to 6.4 kHz;
  b. Material sample size requirements: 29 mm diameter, 200 mm max sample length.

#### 2.2.2. Testing with Larger Sample Size

The results of the preliminary study informed the second stage of acoustical testing. Substrates that resulted in the structural failure of the samples were omitted. Two of the most promising substrates from the preliminary study were determined for both low-frequency and high-frequency sound absorption. These were SCL and FCL and SCH and FCH, respectively. Six replicates were created for each of the low-frequency samples (SCL and FCL), and 9 replicates were created for each of the high-frequency samples (SCH and FCH). These are listed on Table 2 and Table 3. All replicates’ thicknesses were 38 mm. These samples were each tested again, three times, using an impedance tube following the same standard (ASTM E1050-12).

#### 2.2.3. Statistical Analysis

There are usually six frequencies used to determine whether a material is sound absorbing. These are: 125 Hz, 250 Hz, 500 Hz, 1000 Hz, 2000 Hz and 4000 Hz. If the average sound absorption coefficient to the above-stated six frequencies α is bigger than 0.2, the material is called a sound absorbing material [28]. For comparison of the two selected sample groups, the sound absorption coefficients at the following low-frequency levels are used for the 100 mm samples: 125 Hz, 250 Hz, 500 Hz, and 1000 Hz; additionally, the sound absorption coefficients at the following high-frequency levels are used for the 29 mm samples: 2000 Hz and 4000 Hz. The mean sound absorption coefficients of the sample groups at the given frequency levels were compared using the Mann–Whitney U test with the SPSS software (IBM Corp. Released 2015. IBM SPSS Statistics for Windows, Version 23.0. IBM Corp: Armonk, NY, USA). The Mann–Whitney U test was used to determine whether there is a difference in the dependent variable for two independent groups and to compare whether the distribution of the dependent variable is the same for two groups [29]. A *p*-value of <0.05 was considered statistically significant.

### 2.3. Paneling Experiments

**Figure 5:**
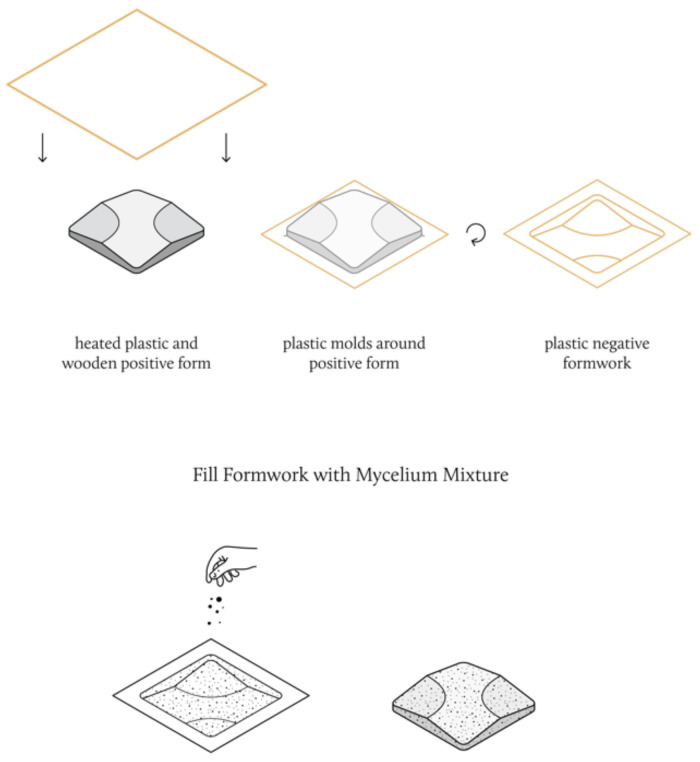
Acoustic Panel Fabrication Diagram.

Initial experiments were conducted regarding the design and fabrication of acoustic panels using the best-performing materials presented in this study. As seen in Figure 5, the fabrication of the panels was performed by first CNC-milling a positive wooden form of 380 mm × 380 mm × 50 mm, and then thermoforming the wooden form with PVC sheets to create a reusable plastic negative formwork. This formwork was then filled with a fine cardboard substrate mixture, and panels were grown using the same procedure presented in Section 2.1.

## 3. Results

### 3.1. Physical Characteristics of the Cultivated Mycelium-Based Composites

A visual inspection was conducted to determine initial growth conclusions. This first analysis was useful in determining which mycelium mixtures were unsuitable for the impedance tube tests and therefore unsuitable as acoustic paneling materials.

#### 3.1.1. Warpage

The materials, once dried, shrank and warped from their original form, as seen in Figure 2d. The material with the most warpage was the Sample FPL (fine paper), and the material with the least warpage was the Sample EML (Ecovative mixture), followed by the Sample FCL (fine cardboard), and the Sample FNL (fine newsprint). In order to ascertain accurate test results from the impedance tube, the sample surface must be flat. As a result, the formworks were made larger than the sample size, so the inaccuracy due to warpage was minimized. For the use of mycelium-based composites as acoustic panels, warpage becomes a challenge for form-to-performance accuracy and mounting purposes. Further research is necessary to predict warpage for specific mycelium mixtures. One possible solution could be to add weights to the corners of the materials as they dry.

#### 3.1.2. Structural Integrity

**Figure 6:**
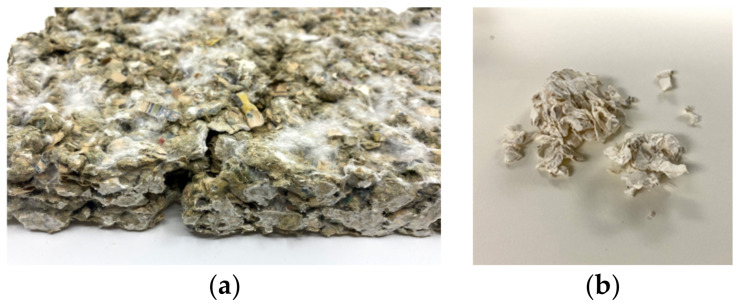
Structural Integrity of Mycelium Samples: (**a**) FNL (**b**) SPH.

The structural integrity of the composite material largely relies on the structure of the substrate material and how well mycelium can grow throughout the substrate. The Sample FNL (fine newsprint) did not hold together once dried, cracked, and crumbled, as seen in Figure 6a. A possible explanation for the deterioration is the structure of the fine newsprint. When pulverized, the newsprint became very fine dust, while the pulverized paper and cardboard maintained more of their structure. After shaping the materials using industrial tools, the materials’ structural integrity was affected, as the fungal skin is a large component holding the material together. The Sample SPH (shredded paper), once cut with a saw, could not yield accurate results in the impedance tube because of its structural integrity, as seen in Figure 6b. Therefore, no further tests were conducted with the FNL, FNH, SPL, and SPH Samples.

Once all the samples were cut open, the inner growth of the samples was analyzed. The samples were observed to have higher mycelial growth on the outer surface and less mycelial growth internally. This could be related to the absence of light and air and the heat produced by mycelium during growth [15]. While the shaping of the samples was necessary for the impedance tube tests, it is worth noting that for the purpose of acoustic panels, shaping/cutting the samples negatively affects their durability and structural integrity.

It is possible that a combination of different substrate materials would lead to more mycelium growth and stronger material. Further research can be conducted to assess the mechanical and physical characteristics of mycelium-based composites grown on different combinations of paper-based waste substrates.

### 3.2. Sound Absorption of the Cultivated Mycelium-Based Composites

#### 3.2.1. Results of the Preliminary Acoustic Absorption Testing

The results of the impedance tube for each sample were recorded and graphed. For each material mixture, two replicates grown in a single formwork were tested, and their results were averaged. It is important to note that the surface of the samples varied depending on the growth of the mycelium. These tests were useful in determining which mixtures performed better than others and informed the second stage of acoustical testing with additional replicates.

**Figure 7:**
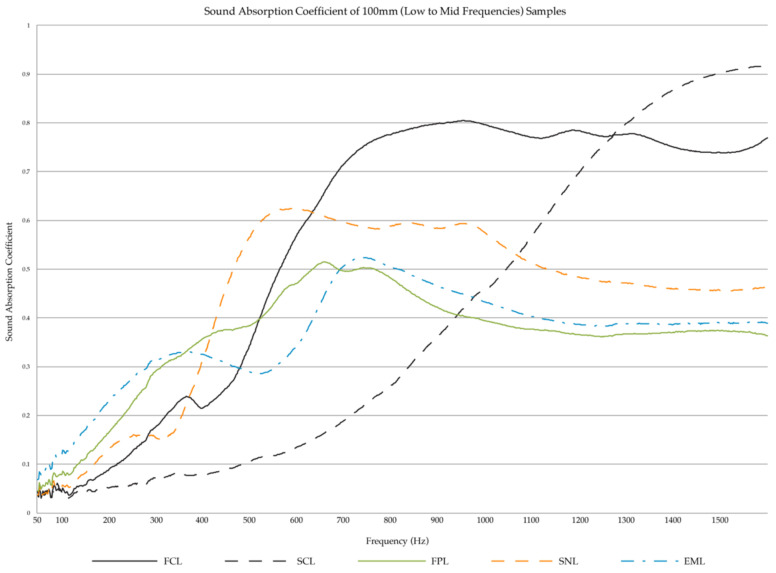
Sound Absorption Coefficient of low-frequency samples (100 mm).

Of the low to mid-frequency samples, the fine cardboard samples (FCL) showed the best absorption, as can be seen in Figure 7, though none of the samples showed very high absorption in the low-frequency range (50 Hz to 500 Hz). It was noted that the sound absorption results shown do not include the effect of an air gap behind the material. The introduction of an air cavity between the material and the rigid backing surface can increase the sound absorption performance at low frequencies [30]. Of the mid-range frequencies (500 Hz to 2 kHz), the fine cardboard samples FCL had the best acoustic absorption performance.

**Figure 8:**
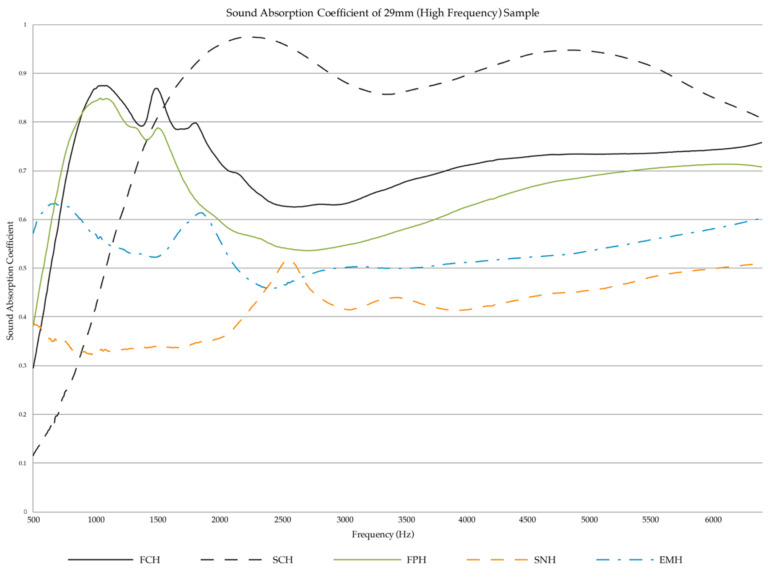
Sound Absorption Coefficients of high frequency samples (29 mm).

Of the high-frequency samples, the shredded cardboard samples (SCH) had the highest sound absorption from the 2 kHz to 6.4 kHz frequency range, as can be seen in Figure 8. Samples SCH is followed by the fine cardboard samples (FCH), then the fine paper samples (FPH). The lowest absorption is from the shredded newsprint samples (SNH). EMH does not show to be a successful absorber.

The graphs in Figure 7 and Figure 8 display the results for the 100 mm and 29 mm samples, respectively. The first graph (Figure 7) represents the materials’ absorption from the 50 Hz to 1.6 kHz frequency range. The second (Figure 8) is from 500 Hz to 6.4 kHz.

#### 3.2.2. Results of the Acoustic Absorption Testing with Larger Sample Size

**Figure 9:**
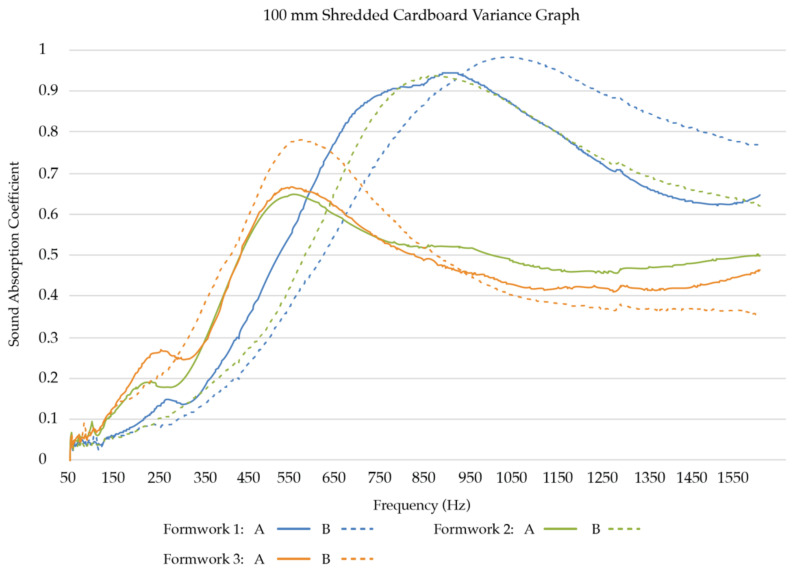
Sound Absorption Coefficients of SCL replicates (100 mm).

**Figure 10:**
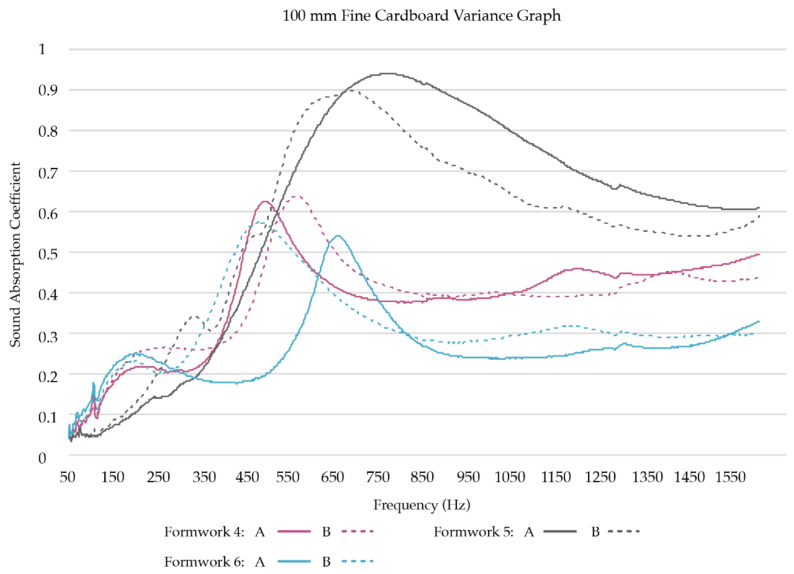
Sound Absorption Coefficients of FCL replicates (100 mm).

*Low-frequency sound absorption coefficients:* To obtain a better understanding of how the two best-performing samples in low-frequency sound absorption, SCL and FCL, compare with each other, we created six replicates for each sample group and tested their acoustic absorption. Three formworks were filled for each substrate mixture, resulting in two replicates per formwork, thus six replicates per substrate mixture (Table 2). Figure 9 presents the test results of the six SCL replicates grown in three separate formworks. Figure 10 presents the test results of the six FCL replicates grown in three separate formworks.

**Table 2:** Samples Tested for Low-Frequency Sound Absorption.

Abbr.	Sample	Formwork Number	Number of Replicates	Sample Size
SCL	Shredded Cardboard Low freq.	1–2–3	6	100 mm
FCL	Fine Cardboard Low freq.	4–5–6	6	100 mm

Of the low to mid-frequency samples, the shredded cardboard samples (SCL) follow two general trends. Half of the replicates’ sound absorption coefficients peak between 450 Hz and 650 Hz and then begin to drop, while the other half has a much higher absorption rate, and the absorption peak shifts to between 750 Hz and 1050 Hz. Of the mid-range frequencies (500 Hz to 2 kHz), SCL performs well, with half of the replicates reaching over a 0.9 sound absorption coefficient at some frequency.

The fine cardboard samples (FCL) similarly follow two general trends in the low to mid-frequency ranges. Four of the replicates’ sound absorption coefficients peak between 400 Hz and 700 Hz, then drop and remain constant, while the other two have a much higher absorption rate, and the absorption peak shifts to between 550 Hz and 850 Hz. Of the mid-range frequencies (500 Hz to 2 kHz), some of the FCL also perform well, with two of the replicates reaching a 0.9 sound absorption coefficient at some frequency.

As can be seen in Table 4, the test results show that in the selected low to mid frequencies (125 Hz, 250 Hz, 500 Hz, 1000 Hz), the sound absorption trends of both low-frequency sample groups (SCL and FCL) are statistically similar (*p* > 0.05).

**Figure 11:**
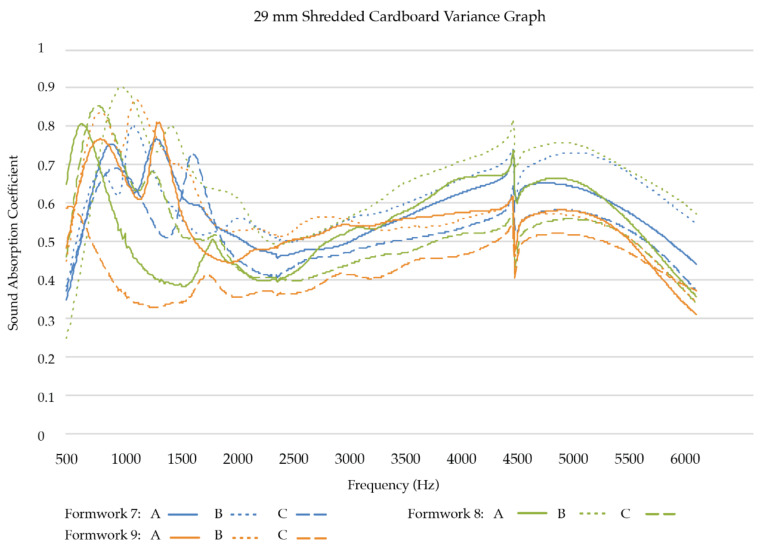
Sound Absorption Coefficients of SCH replicates (29 mm).

**Figure 12:**
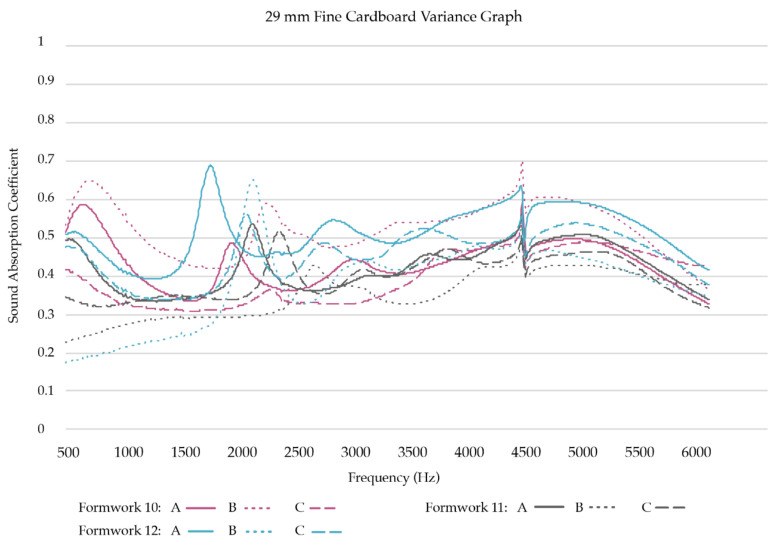
Sound Absorption Coefficients of FCH replicates (29 mm).

*High-frequency sound absorption coefficients:* To obtain a better understanding of how the two best performing samples in high-frequency sound absorption, SCH and FCH, compare with each other, we created nine replicates for each sample group and tested their acoustic absorption. Three formworks were filled for each substrate mixture, resulting in three replicates per formwork, thus nine replicates per substrate mixture (Table 3). Figure 11 presents the test results of the nine SCH replicates. Figure 12 presents the test results of the nine FCH replicates.

**Table 3:** Samples Tested for High-Frequency Sound Absorption.

Abbr.	Sample	Formwork Number	Number of Replicates	Sample Size
SCH	Shredded Cardboard High freq.	7–8–9	9	29 mm
FCH	Fine Cardboard High freq.	10–11–12	9	29 mm

Of the high-frequency samples, the shredded cardboard samples (SCH) follow a single trend. The samples generally peak between 500 Hz and 1.5 kHz, dip and then gradually rise again. The samples have a medium to high absorption rate from 500 Hz to 1.5 kHz and 4 kHz to 5.5 kHz. The fine cardboard samples (FCH) also follow a single trend. However, as can be seen in Table 4, in the selected high frequencies (2000 Hz, 4000 Hz), high-frequency sample groups (SCH and FCH) have different sound absorption trends (*p* = 0.019 and *p* = 0.011, respectively). Shredded cardboard samples (SCH) had better sound absorption performance than fine cardboard samples (FCH).

**Table 4:** Sound absorption coefficients of shredded and fine cardboard samples (mean ± Standard Deviation).

	Frequencies (Hz)	Shredded Cardboard (SCL + SCH)	Fine Cardboard (FCL + FCH)	*p*-Value
Low Freq.	125	0.0698 ± 0.02	0.1218 ± 0.05	0.070
	250	0.1609 ± 0.07	0.2074 ± 0.04	0.240
	500	0.5116 ± 0.17	0.5096 ± 0.15	0.810
	1000	0.6891 ± 0.26	0.4697 ± 0.23	0.070
High Freq.	2000	0.4934 ± 0.07	0.3949 ± 0.08	0.019
	4000	0.5731 ± 0.07	0.4703 ± 0.06	0.011

### 3.3. Mycelium-Based Acoustic Panel Prototypes Cultivated with Fine Cardboard Substrates

**Figure 13:**
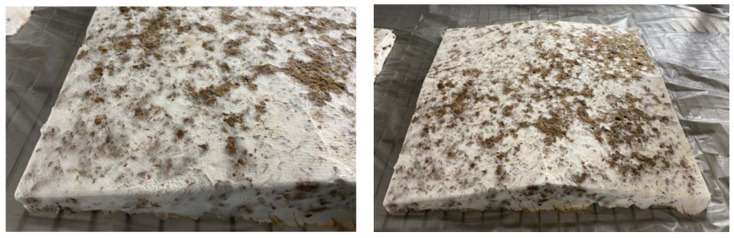
Acoustic panel prototype cultivated with Fine Cardboard substrate.

The results of the acoustic panel prototypes revealed that mycelium growth is still consistent even in larger formworks (Figure 13). However, the durability of the material proves to be a problem on a larger scale. After dying and handling, the edges of the panels began to show signs of deterioration. In order to ensure durability with the fine cardboard material, additional support may be necessary. This could potentially be remedied with additional substrate materials, internal support, or external backing.

**Figure 14:**
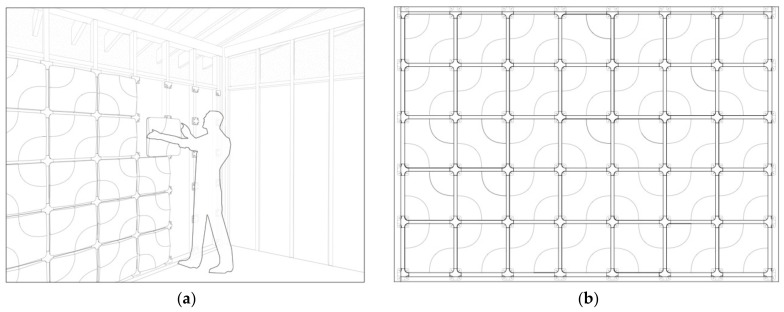
Parametric acoustic panel wall prototype design: (**a**) Acoustic panel installation illustrated (**b**) Example wall configuration.

Concurrently with panel fabrication, a customizable panel system was generated using parametric modeling software (Rhinoceros 3D, Version 7.0. Robert McNeel & Associates, Seattle, WA, USA). Figure 14 shows a custom acoustic wall configuration generated using this system and illustrates how the panel configuration can be altered. The use of the parametric system aids in random wall configurations. Using a three-dimensional truchet tile in the parametric system allows for a number of wall configurations with only one panel, thus reducing the need for different formworks.

## 4. Discussion

The results of this study indicate that mycelium-based composites grown on waste shredded and fine cardboard show potential as sound-absorbing materials, specifically in the mid to high-frequency ranges. Shredded cardboard samples (SCH) slightly outperform fine cardboard samples (FCH) in high-frequency ranges.

### 4.1. Comparison to Commercial Sound Absorbing Materials

The impedance tube test results show that mycelium-based composites cultivated on shredded cardboard and fine cardboard can both be considered sound-absorbing materials (α > 0.2) and have the potential to compete with the performance of synthetic sound absorbers (Table 5). The sound absorbing coefficients of three types of commercially available synthetic sound absorbing products made with fiberglass, polypropylene, and plaster were compared with the fine cardboard samples (FCL + FCH) and shredded cardboard samples (SCL + SCH). The comparison revealed that the fiberglass insulation board shows better sound absorption than both sample groups in all the frequencies except 125 Hz. When compared with the polypropylene product, both sample groups have better sound absorption at low frequencies (125 Hz, 250 Hz, 500 Hz). Compared with plasterboard, one of the most common interior wall finishes, the absorption coefficients of both samples are significantly higher in the mid to high-frequency ranges. These comparisons are helpful in discussing the potential of the two sample groups as sound absorbers. For more accurate comparisons, commercially available sound-absorbing materials need to be tested by the authors for each frequency range using the same testing model.

### 4.2. Effects of Surface Texture and Porosity on Sound Absorption

**Figure 15:**
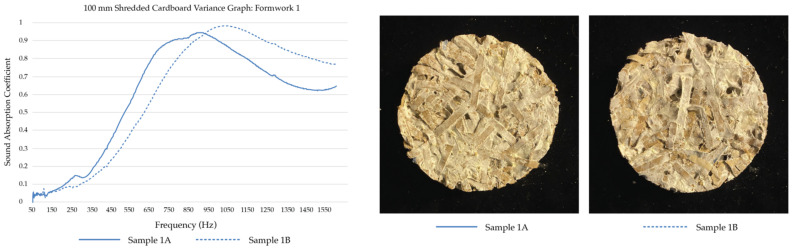

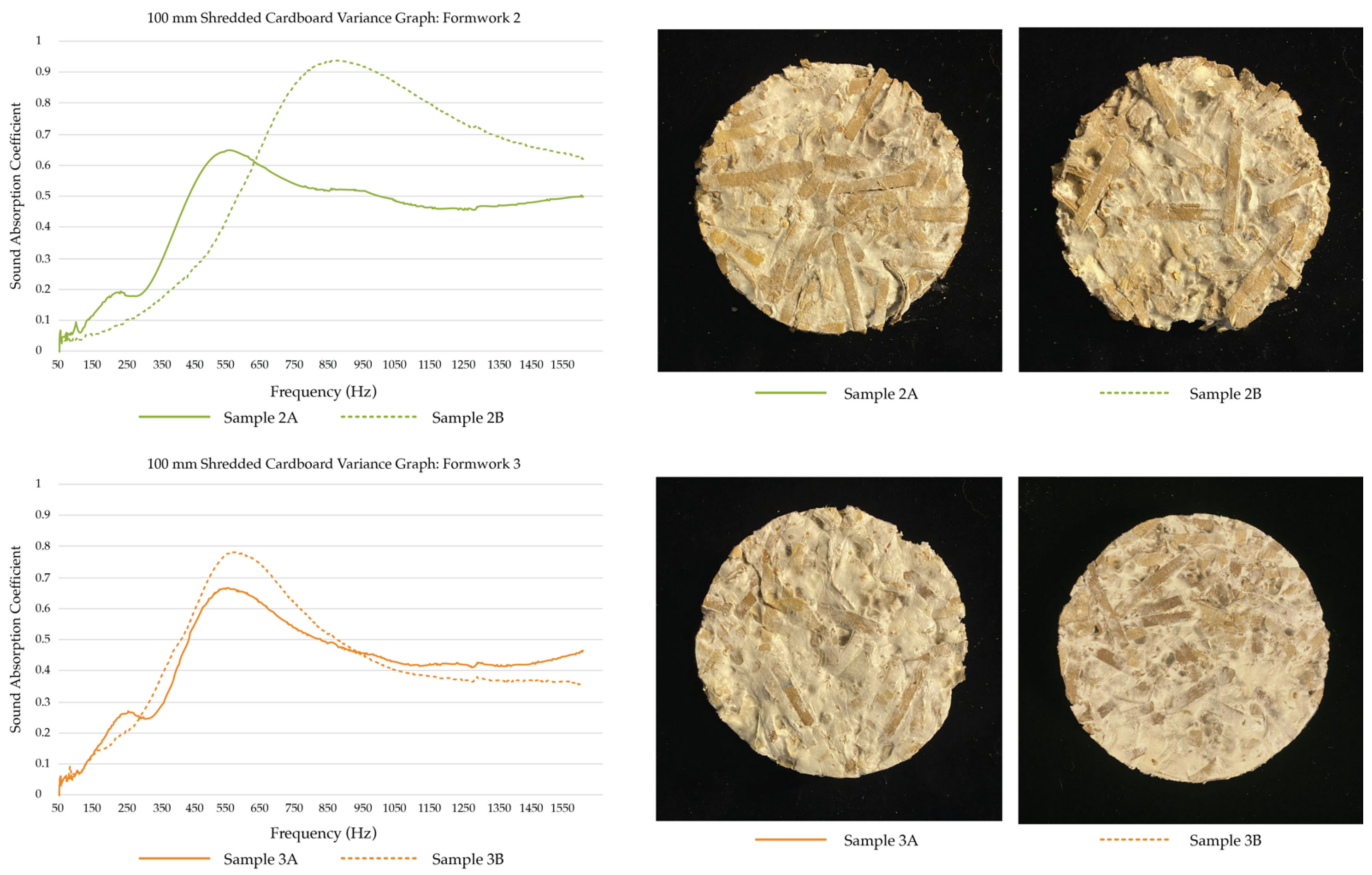
Sound Absorption Coefficient Graphs and the Shredded Cardboard Samples Tested. A visual inspection to compare surface texture and porosity with the respective material performance.

The results of the impedance tube tests revealed significant variances between replicates of the same material (see Table 4). This is hypothesized to be the result of inconsistent mycelia growth, the size of the substrate material, and the random substrate filling technique. The following graphs in Figure 15 present the sound absorption coefficients of SCH replicates cultivated within three different formworks, alongside close-up images of the replicates cultivated within the same formwork. A visual inspection revealed that the replicates with more bumps and pores at the surface have higher sound absorption coefficients; however, further tests are needed to validate this hypothesis.

### 4.3. Limitations and Strengths of the Study

There are two main limitations to this study. The first limitation addresses Section 4.1. The data for the commercially available synthetic sound absorbers were collected from the existing literature [31,32,33]. While the sound absorption coefficients for these materials are validated numbers provided in their data sheets, to be able to ensure accurate results and have a meaningful comparison, commercially available sound absorbing materials need to be tested for each frequency range using the same testing model, with samples that have the same material thickness, density, and porosity.

The second limitation addresses Section 4.2. As can be seen in Table 4, the sound absorption coefficients for both sample groups in 500 Hz and 1000 Hz frequencies show significant variances. This limitation can be overcome by creating larger subgroups within each sample group through visual inspection of the replicates and testing these subgroups’ sound absorption coefficients independently.

The strength of the study was initially performing preliminary tests with multiple waste paper-based samples. This enabled accurately deciding which substrates fit in the testing model and eliminating the ones that did not work.

## 5. Conclusions

Of the tested samples from the preliminary acoustic tests, the shredded and fine cardboard-based samples show the best acoustic performance. In addition to this, the fine newsprint and shredded paper substrates are not considered to be applicable for paneling purposes due to their (lack of) structural integrity. Due to these findings, the shredded and fine cardboard samples were regrown with larger sample size and tested again. The results show that both shredded and fine cardboard-based mycelium composites do show potential as sound absorbing materials, with shredded cardboard samples slightly performing better in high-frequency sound absorption. However, the inherent nature of bio-fabricated materials causes a variance in performance, even between samples of the same material.

The next steps of this research are to investigate how material thickness, density, and porosity affect sound absorption of shredded and fine cardboard-based mycelium composites. This will be performed by cultivating additional replicates and creating larger subgroups within each sample group by controllably varying their material thickness, density, and porosity. Along with their sound absorption properties, their mechanical properties (compression, bending, torsion, and tension and impact damping) and morphological characteristics (i.e., pore size, porosity, density), as well as the growth mechanisms of mycelium, will be studied. The main objective is to understand how the growth of mycelium at microscopic levels, the morphological characteristics at both mesoscopic and macroscopic levels, and the acoustic absorption performance of the composites interact with one another. Another follow-up study could be to test various commercially available synthetic acoustic absorbers using the same testing model, with samples that have the same material thickness, density, and porosity as the mycelium-based sample groups. This would enable a more thorough comparison of mycelium-based composites’ acoustic absorption performance with synthetic absorbers.

Once a holistic understanding and more comprehensive data about the composites’ acoustic, mechanical, and morphological characteristics are gathered, the next steps involve the applications of the shredded and fine cardboard-based composites as acoustic paneling. The material itself, though sound absorbing, has physical limitations such as structural integrity and warping when cultivated on larger scales. More experiments must be conducted to ensure the durability of the material. Concurrently with durability assessments, analyses regarding form-to-performance will be conducted. These experiments will be used to determine how the form of the acoustic panels affects the sound absorption performance. Therefore, full-scale prototypes will be built and tested alongside computer simulation models in reverberant chambers. These results will inform parametric iterations of panel systems.

Incorporating mycelium-based composites into architectural systems is significant because of their ability to reduce waste generated and energy consumed during material manufacturing compared to conventional building materials. Mycelium-based composites recycle waste materials for growth, require little energy to manufacture, and completely decompose at the end of their product life. This research is relevant in order to establish protocols for material use and implementation within acoustic systems.

## Figures and Tables

**Table 1:** Cultivated Samples.

Sample	Abbr.	Substrate	Substrate Treatment	Sample Size
Shredded Cardboard High freq.	SCH	cardboard	shredded	29 mm
Shredded Cardboard Low freq.	SCL	cardboard	shredded	100 mm
Fine Cardboard High freq.	FCH	cardboard	pulverized	29 mm
Fine Cardboard Low freq.	FCL	cardboard	pulverized	100 mm
Shredded Paper High freq.	SPH	paper	shredded	29 mm
Shredded Paper Low freq.	SPL	paper	shredded	100 mm
Fine Paper High freq.	FPH	paper	pulverized	29 mm
Fine Paper Low freq.	FPL	paper	pulverized	100 mm
Shredded Newsprint High freq.	SNH	newsprint	shredded	29 mm
Shredded Newsprint Low freq.	SNL	newsprint	shredded	100 mm
Fine Newsprint High freq.	FNH	newsprint	pulverized	29 mm
Fine Newsprint Low freq.	FNL	newsprint	pulverized	100 mm
Ecovative Mixture High freq.	EMH	undisclosed	undisclosed	29 mm
Ecovative Mixture Low freq.	EML	undisclosed	undisclosed	100 mm

**Table 5:** Sound Absorption Coefficients comparing commercial sound absorbing materials with the fine cardboard samples (FCL + FCH) and shredded cardboard samples (SCL + SCH).

Product/Sample	Octave Band Center Frequencies, Hz						Average
	125	250	500	1000	2000	4000	α
Fine Cardboard Samples (FCL + FCH)	0.12	0.21	0.51	0.47	0.40	0.47	0.36
Shredded Cardboard Samples (SCL + SCH)	0.07	0.16	0.51	0.69	0.49	0.57	0.42
Type 706 Series Fiberglas™ Insulation Board (Fiberglass) [31]	0.01	0.22	0.67	0.97	1.05	1.06	0.66
Quiet Board™ Acoustic Panel (Polypropylene) [32]	0.05	0.06	0.21	0.8	0.65	0.75	0.42
Plasterboard (1/2″ paneling on studs) [33]	0.29	0.1	0.06	0.05	0.04	0.04	0.10

## Data Availability

Data are available upon request.
